# Supplementary material for: The Heterochromatin protein 1 is a regulator in RNA splicing precision deficient in ulcerative colitis
Source: Nat Commun. 2022 Nov 18;13:6834. doi: 10.1038/s41467-022-34556-3 (PMC9674647; doi:10.1038/s41467-022-34556-3)
Supplement: Supplementary file 1 — Supplementary Information [file 41467_2022_34556_MOESM1_ESM.pdf]

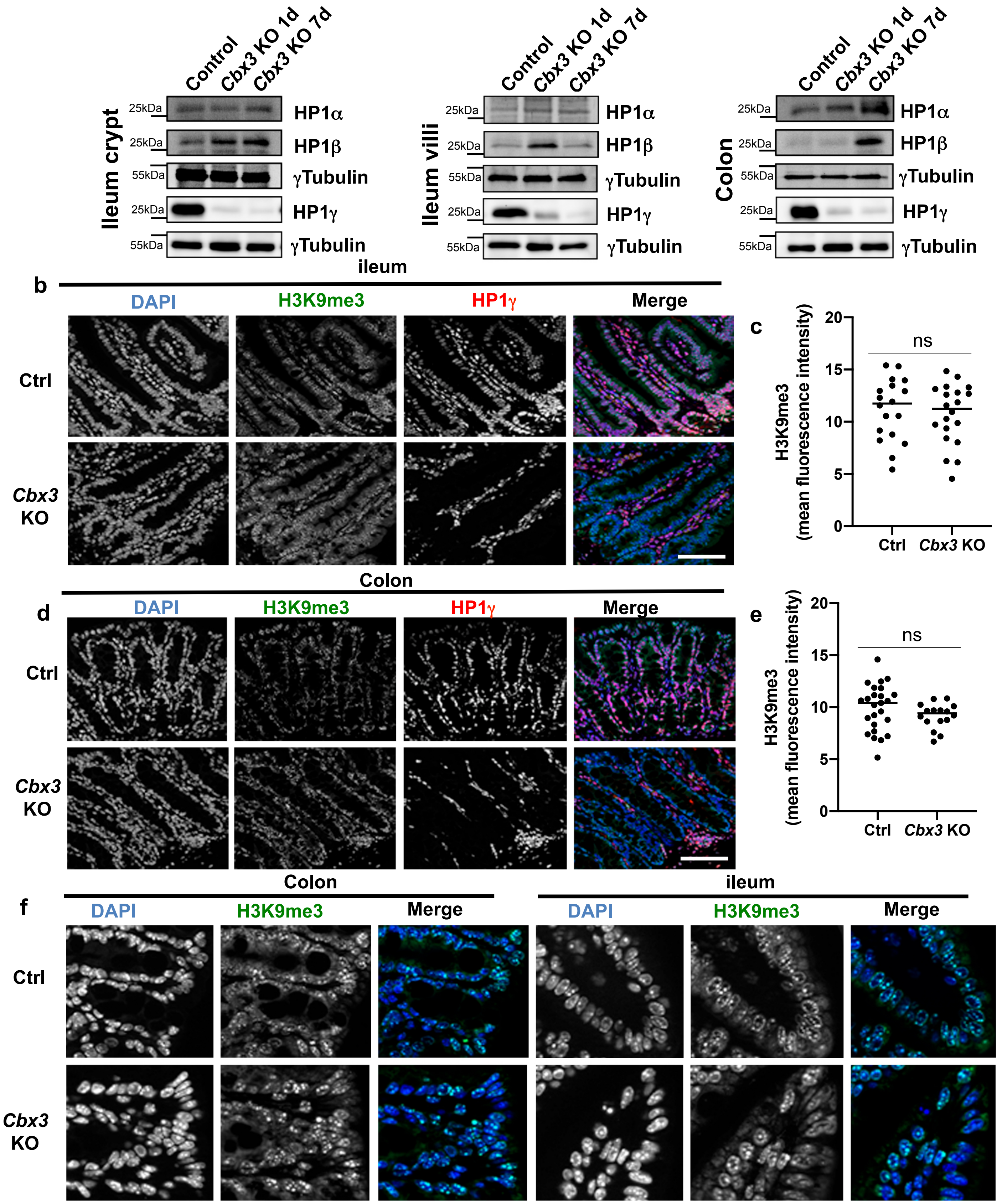

Supplementary Figure 1: Validation of the *Cbx3* KO mice model (**a**) Western blot analysis: time course of HP1 isoforms expression upon *Cbx3* knock-down 1-day and 7-day post-tamoxifen gavage in the crypt, villi and colon epithelia, representative of 3 separate experiments (**b, f**) Immunostaining HP1γ and H3K9me3 in ctrl (vehicle treated) and *Cbx3* KO mice, Scale bar: 80µm and in (**c, e**) imageJ quantification of the H3K9me3 signal in ileum (**c**) and colon (**d**) n=5 mice/group, each point shows the mean fluorescence intensity per nucleous, expressed in arbitrary units, two-sided Student's t-test Source data are provided as a Source Data file.

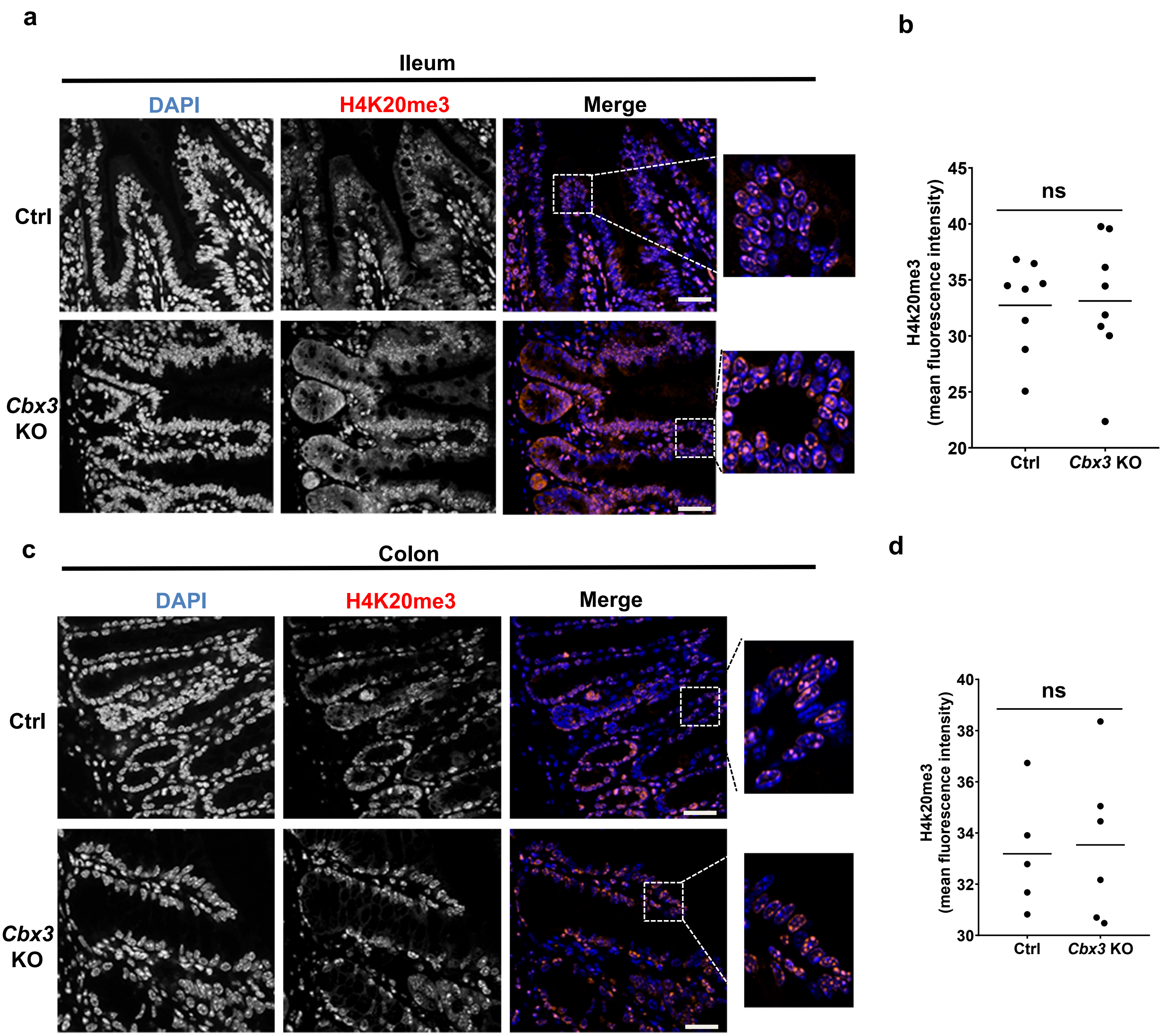

Supplementary Figure 2: Immunostaining of H4K20me3 in the *Cbx3* KO mice model
(**a,c**) Representative immunostaining o f H4k20me3 in ctrl (vehicle treated) and *Cbx3* KO mice and in (**b,d**) imageJ quantification of the H4k20me3 signal in ileum (**b**) and colon (**d**) epithelia in at least n=5 mice/group of 15 fields per condition, where each point represents the mean fluorescence intensity per nucleus in one individual animal, expressed in arbitrary unit. Scale bar: 50μm, 20μm in insert. Two-sided Student's t-test. Source data are provided as a Source Data file.

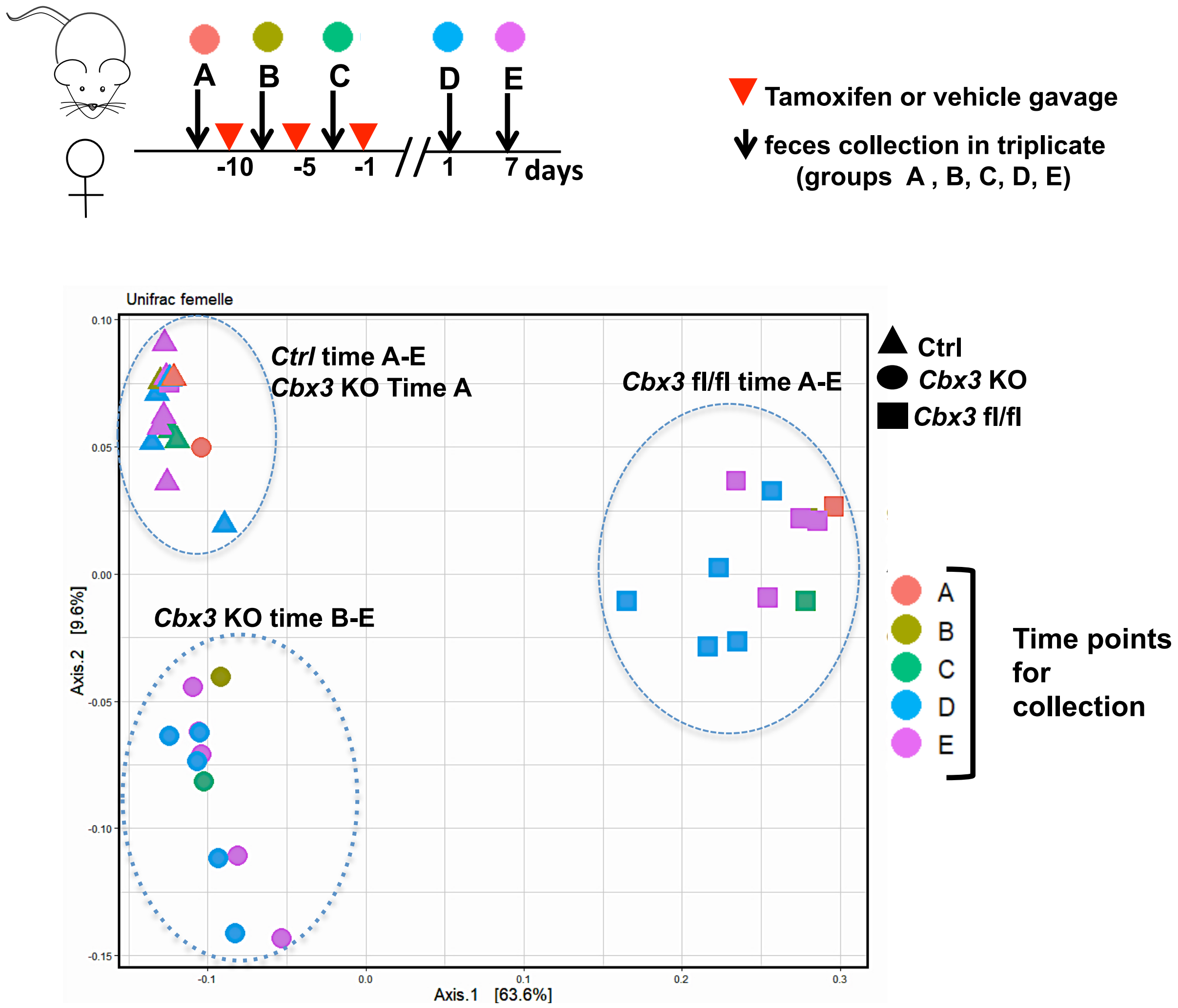

Supplementary Figure 3: Temporal evolution of the Beta diversity in Villin-Cre *Cbx3* KO female mice before (group A) and after treatments (Vhc or Tamox) (groups B-E): (a) Sheme illustrating the fecal sample collection method before and after treatments in the Ctrl (n=4), *Cbx3* KO (n=4)and *Cbx3* fl/fl (n=3) mice (**b**) Beta diversity showing a shift only in the *Cbx3* KO mice. Tamoxifen treatment did not impact beta diversity in the *Cbx3* fl/fl mice that do not expressed the CRE recombinase. Source data are provided as a Source Data file.

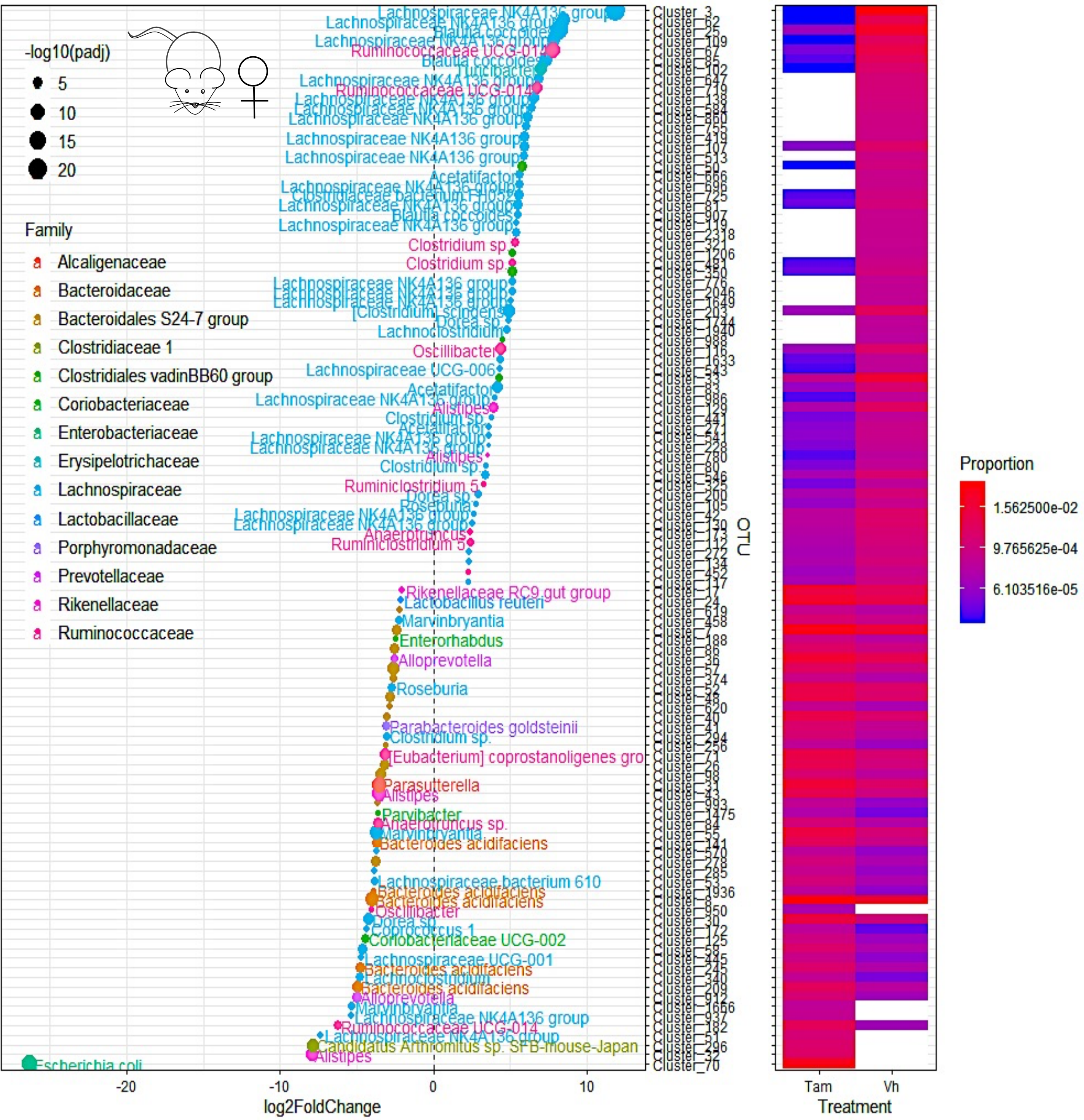

Supplementary Figure 4: Heat map representing the differentially abundant OTUs between tamoxifen (TAM) and Vehicle (Vh) treated *Cbx3* KO in female mice feces (n=4 in each treated group) with fold change > 2 and significant (P < 0.05) using DESeq2. For the graph on the left, each OTU is represented by a dot and colored according to its taxonomic classification at the family level. Taxonomy at the genus or species level is also indicated, when available, next to each OUT (also detailed in **Sup Table 5**). A logarithmic scale (log-2) is used for the x axis.

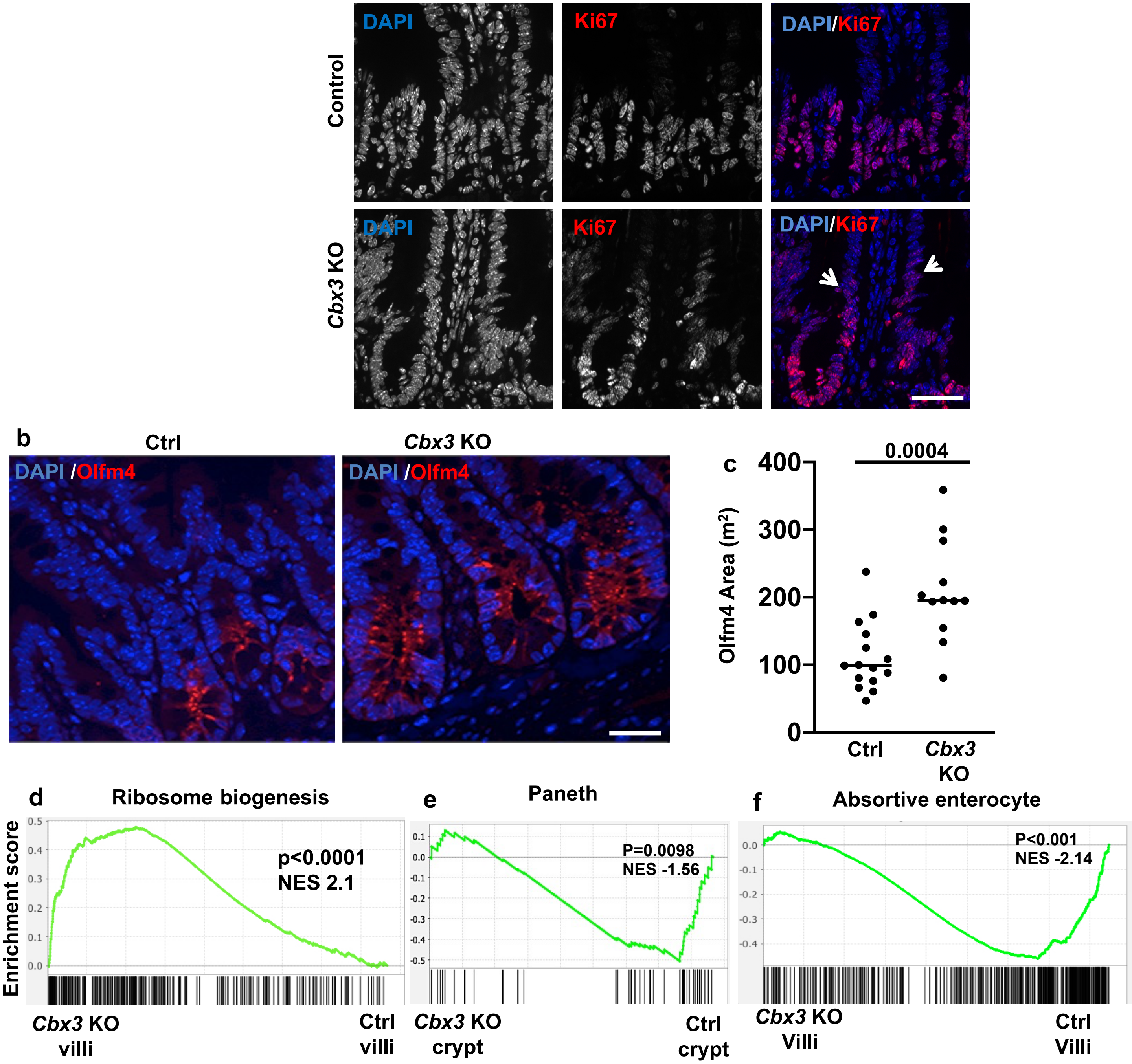

Supplementary Figure 5: Extended Ki67 signal at the crypt villi axis in the Cbx3 KO small intestine: **(a)** Representative immunostaining of Ki67 (red) and Dapi (blue) in control and *Cbx3* KO mice. Arrow head shows aberrant Ki67 signal detected at the base of the villi epithelium. Scale bar: 50µm. Data are representative of at least n=5 mice/condition. **In (b-c) Area of detection of the stem cell mark Olfm4** (**b**) Immunofluorescence with olfm4 antibody (red) and Dapi (blue) of ileal crypt sections from Ctrl and *Cbx3* KO mice, (Scale bar: 50µm) (**c**) Quantification of the Olfm4 expression area. Statistical analysis carried out in n=6 mice, measuring a minimum of 20 fields/mice, Student's *t* test. Two-sided Student's t-test. Source data are provided as a Source Data file.

In (d-f) GSEA analyses: Significant enrichment of the villi *Cbx3* KO transcriptome with ribosomal biogenesis signature (GOBP_RIBOSOME_BIOGENESIS) In **(e-f)** Inverse correlation between *Cbx3* KO and ctrl (control) mice for Paneth and absortive enterocyte signatures (Haber et al, 2017) at the crypt and villi compartments, respectively. Two-sided nominal *P* values were calculated by GSEA.

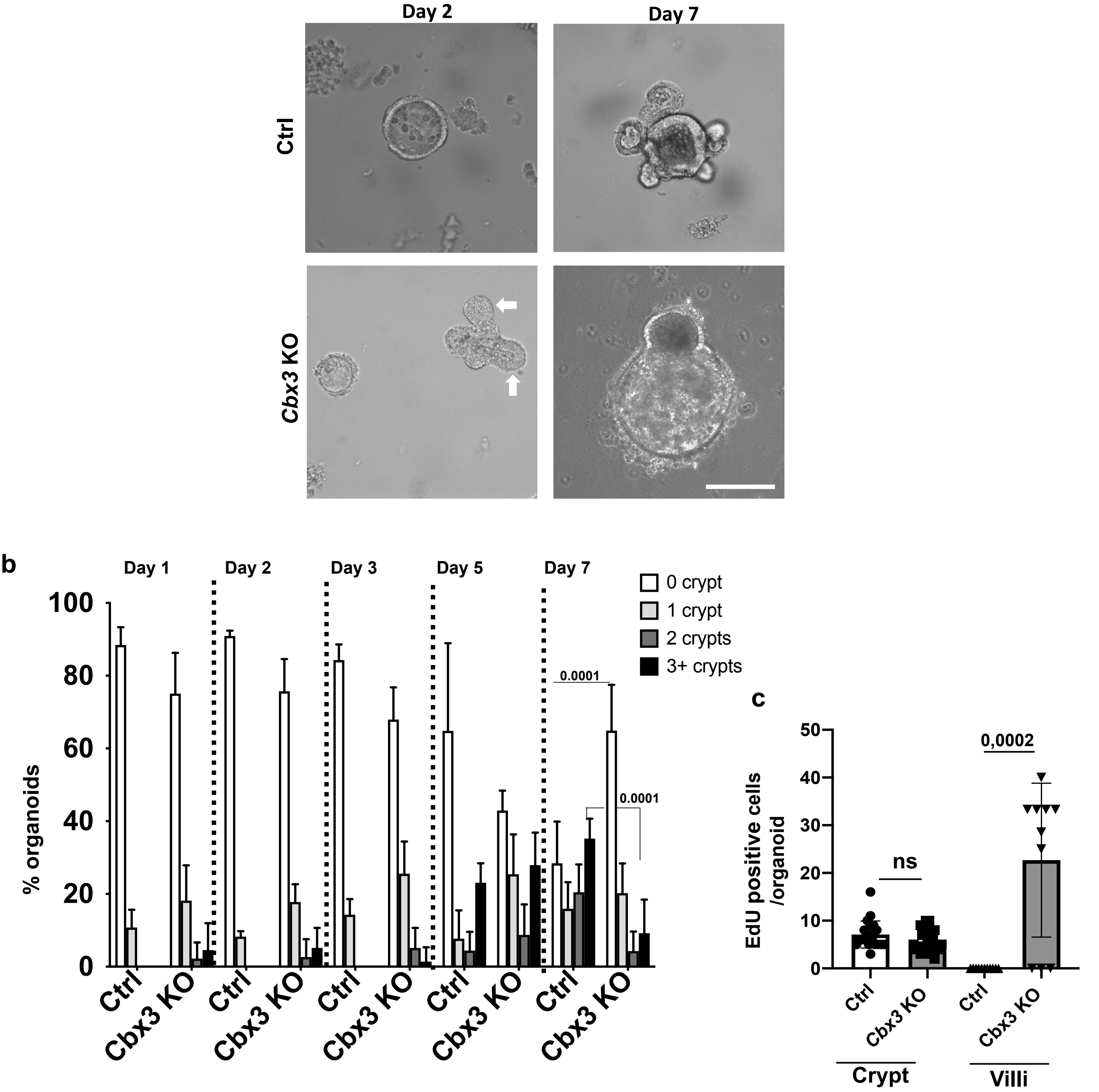

Supplementary Figure 6: *Ex vivo* enteroid 3 D matrigel cultures upon *Cbx3* inactivation: **(a-b)** Time course of organoid budding, (**a**) Representative image, taken by transillumination microscopy. Initial increase in budding formation (white arrows) was not maintained overtime. Scale bar: 80µm. (**b**) Evolution of the number of crypts per organoid over time. At day 7, organoid complexicity (i.e. budding crypt potential) was significantly higher in ctrl, as compared to *Cbx3* KO organoids. Counting was carried out on a minimum of 100 organoids, in three different animals, per condition. Statistical analysis was carried out by one-way ANOVA. Data are presented as mean ± SEM . **In (c)** Quantification of the EdU signal in organoids derived from 3 different animals /group, from a total of n=10-25 organoids/group. Data are presented as the mean ± SEM. Two-sided Student's t-test. Source data are provided as a Source Data file.

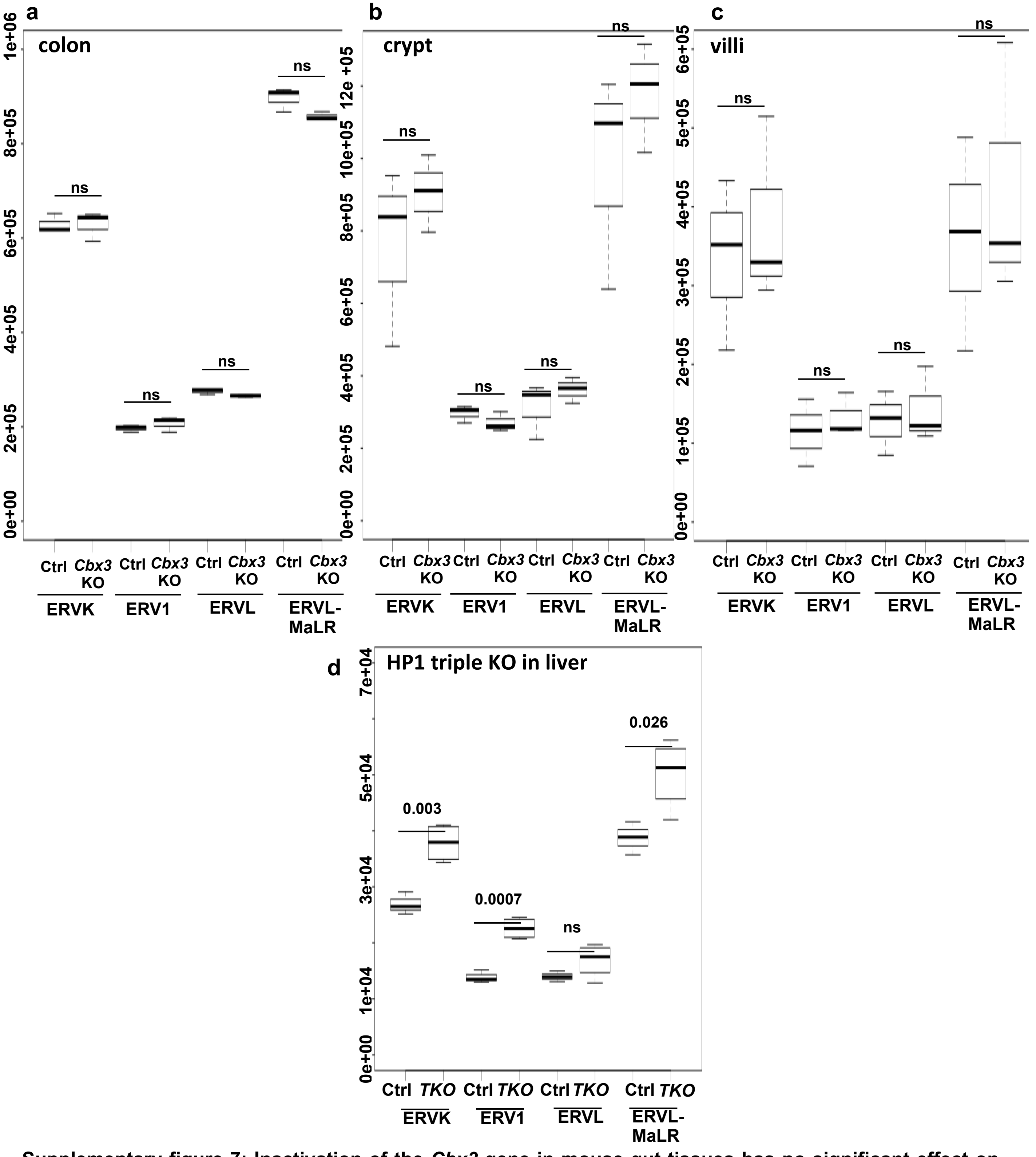

Supplementary figure 7: Inactivation of the *Cbx3* gene in mouse gut tissues has no significant effect on transcription of endogenous retroviruses. Reads mapping to sequences annotated as members of the ERVK, ERV1, ERVL, or ERVL_MaLR families of endogenous retroviruses in mm 10 RepeatMasker were quantifies in the indicated tissues upon inactivation either of *Cbx3* (panels **a**, **b**, and **c**), or of *Cbx1, Cbx3,* and *Cbx5* TKO – panel **d**). Number of samples: n= 3 for WT and *Cbx3* KO in colon, crypts, and villi, and for WT livers, n= 4 for TKO livers). Indicated figures were normalized by the number of reads in each replicate. P values were calculated using an unpaired two–sided Student's t test. Boxplot center indicates the median, bounds of the box indicate the 1st and 3rd quartiles, lower and upper whiskers define minima and maxima. Source data are provided as a Source Data file.

a

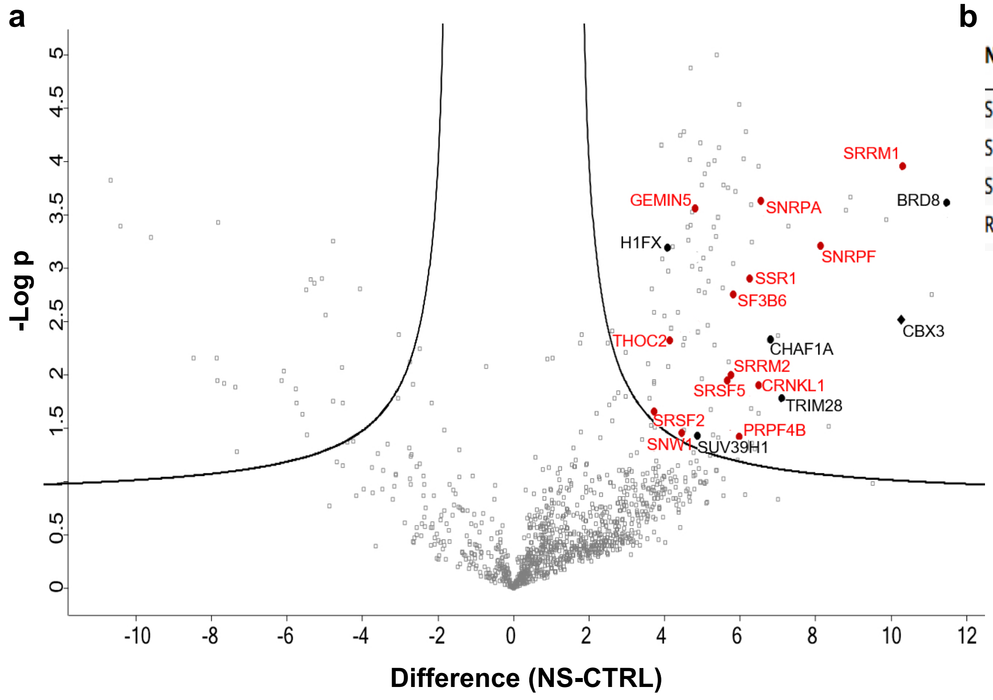

b

Name	P-value	Adjusted p-value
Spliceosome	4.808e-8	0.000004952
Sulfur relay system	0.03815	0.3573
Sulfur metabolism	0.04747	0.3963
RNA transport	0.002130	0.07364

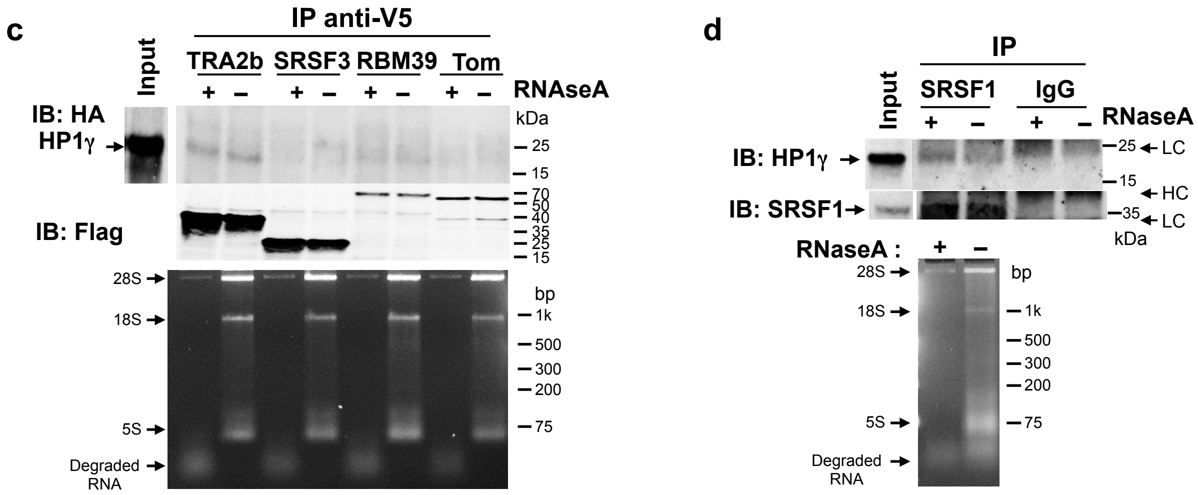

Supplementary Figure 8: Proteomic analysis: (a) Volcano plot representation of proteins immunoprecipitated with anti-HP1γ or IgG (CTRL) antibodies in HeLa cells and identified by mass spectrometry in 3 independent experiments. X-axis reports the difference of the average of the logarithm of Label Free Quantification (LQF) intensities between IgG control and with anti-HP1γ immunoprecipitates. Y-axis reports the negative logarithm of t-test p value. In black: classical HP1 interactors, in red: spliceosome interactors. (**b**) KEGG-pathway analysis on the molecular interactors identified by mass spectrometry (a tot al of 96 hits), pValue and adjusted pValue are calculated within Enrichr using the Fisher exact test.

(**c-d**) HP1γ association with splicing factors resists to RNase treatment, in (**c**) Extracts from HEK293 cells transfected with expression vectors for HA-tagged HP1γ and Flag-V5 tagged indicated splicing factors or Tomato protein (Tom) as negative control were treated (+) or not (-) with RNaseA . Immunoprecipitations were performed with anti-V5 antibodies followed by immunoblot (IB) with the indicated antibodies. Co-immunoprecipitated HP1γ was revealed by anti-HA antibodies

(**d**) Immunoprecipitations were performed with anti-SRSF1 or species-matched non-immune IgG in Hela cells lysates and IB revealed by the indicated antibodies. IgG heavy (HC) and light (LC) chain signals were indicated. Efficiency of RNase treatment was checked by analyzing purified RNA from unbound fraction after immunoprecipitations on BET-stained agarose gel of (bottom panels). Each co-IP was independently performed twice. Source data are provided as a Source Data file.

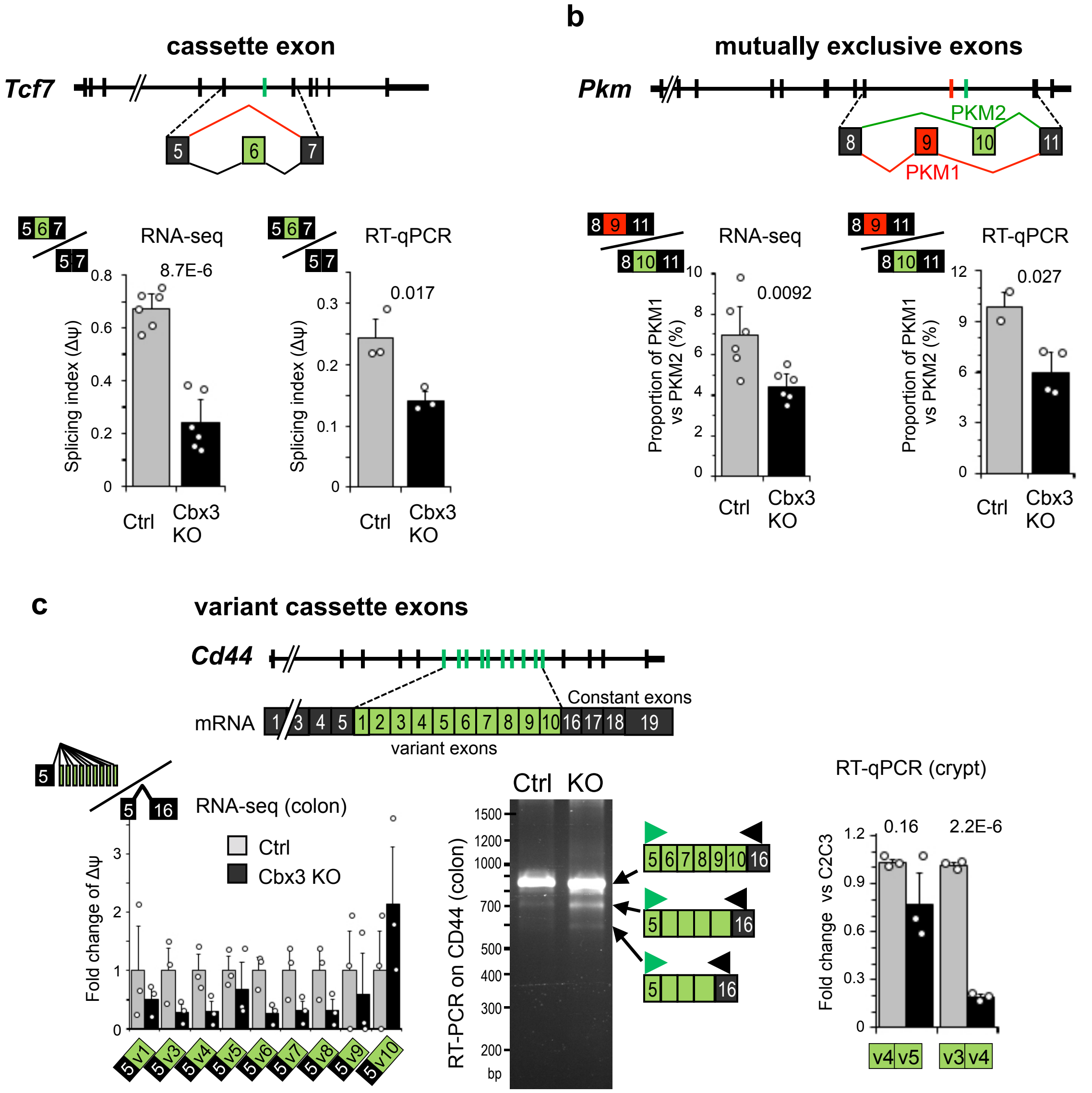

Supplementary figure 9: PCR validation of representative alternative splicing events affected by *Cbx3* inactivation at the indicated genes (**a**) *Tcf7* (cassette exon, crypt epithelium),(**b**) *Pkm* (mutually exclusive exon, colon epithelium) and (**c**) *Cd44* (variant cassette exon, colon and crypt epithelia). Genomic view with schematic of the examined splicing events are shown and the variant exons are in color. The splicing index (Δψ) has been calculated as described in method and validation by RT-qPCR (n=3 mice/group). A representative gel image of RT-PCR products is provided with schematic diagram of the primer positions. In (**c**) the decrease in variant exon inclusion at the *Cd44* gene in the colon is illustrated by the apparition of shorter PCR products corresponding to the expected size when 1 or 2 variant exons are skipped. RT-qPCR on variant exon short junctions performed on crypt RNAs shows a similar trend of decrease variant exon inclusion (n=3 mice / group, right panel). Data are presented as the mean ± SEM. The P-values calculated indicated in graphs with the Student's t test (two-sided). Source data are provided as a Source Data file.

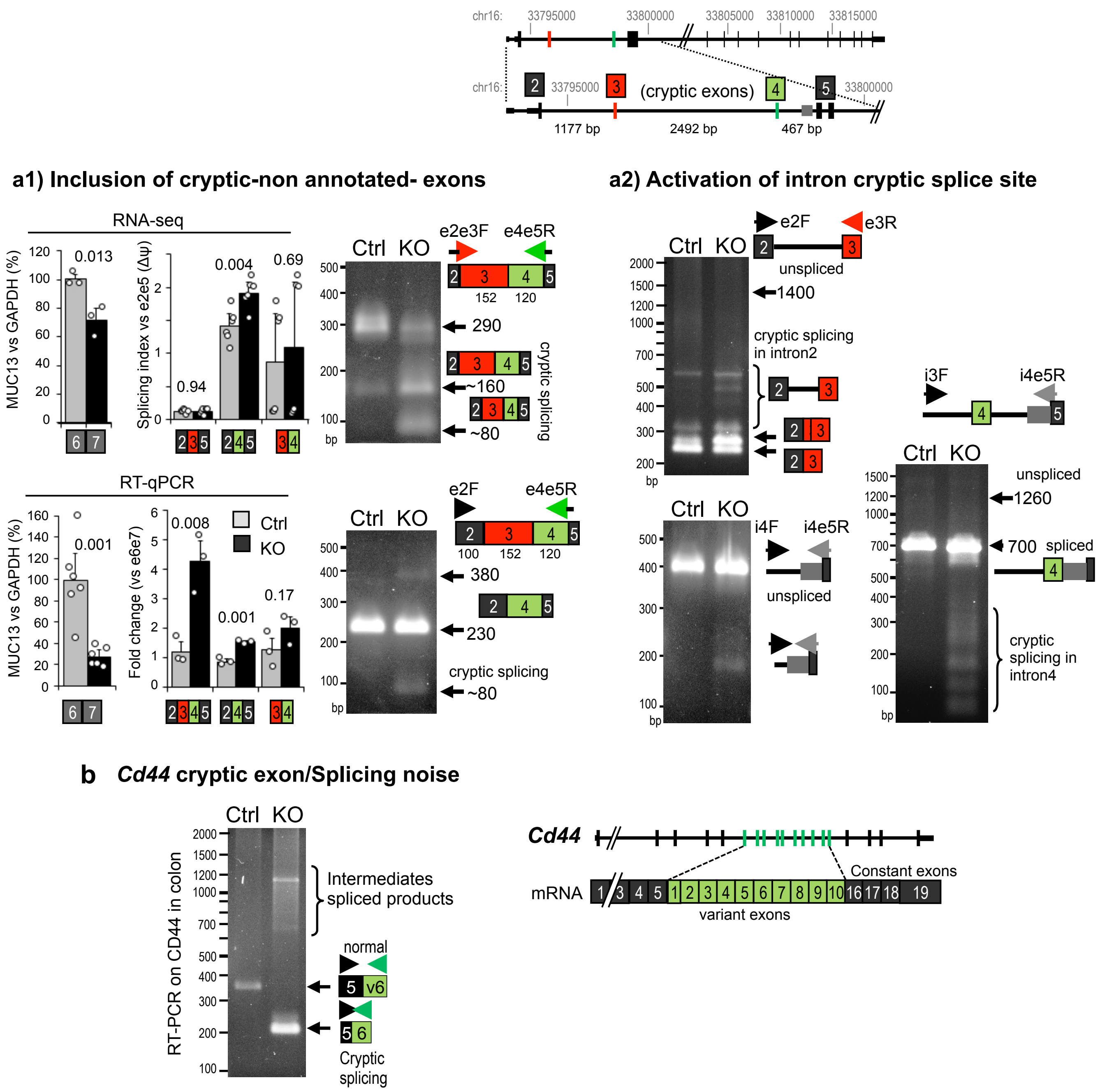

Supplementary figure 10: PCR readout of the splicing noise induced by *Cbx3* inactivation:
(**a**) The *Muc13* gene: boxes representing exons are colored in black when annotated, and in red or green when non-annotated. The expected sizes of introns are indicated.
(**a1**) Increased inclusion of *Muc13* cryptic exons (e3/e4) in the colon epithelium of *Cbx3* KO mice. Left panel: (top left) the overall expression of Muc13 is reduced rather than increased upon *Cbx3* inactivation, as documented by quantification of *Muc13* constant exons relative to GAPDH. (top right and bottom panels) splicing indexes (Δψ) initially obtained from RNA-Seq data were validated by RT-qPCRs (colon epithelium, n=3 mice /group) using primers nested either over a single exon or over exon-junctions as indicated on the schematic. Fold change of variant exons were normalized by the level of annotated exons 6 and 7. Data are presented as the mean±SEM. P-values were calculated using the Student's t test (two-sided)
Right panel: gel electrophoresis of the PCR products. Exon-composition of the species was deduced from their size and from the position of the primers.
(**a2**) Usage of multiple *Muc13* intronic cryptic sites: primers nested on exons (e2:e3) or at intronic positions (i3F, i4F, i4e5R) were used for PCR with prolonged extension times (1 min at 72°C) to favor amplification of long species. Analysis of the PCR products by gel electrophoresis revealed many additional species upon *Cbx3* inactivation.
(**b**) Splicing noise at the *Cd44* gene: PCR with primers nested on *Cd44* constant exon C5 and variant exon v6. Gel electrophoresis documents usage of alternative splice sites within the 2 exons, resulting in a shorter products in *Cbx3* KO. The top of the gel also reveals accumulation of numerous unresolved PCR products in *Cbx3* KO as indicated. Source data are provided as a Source Data file.

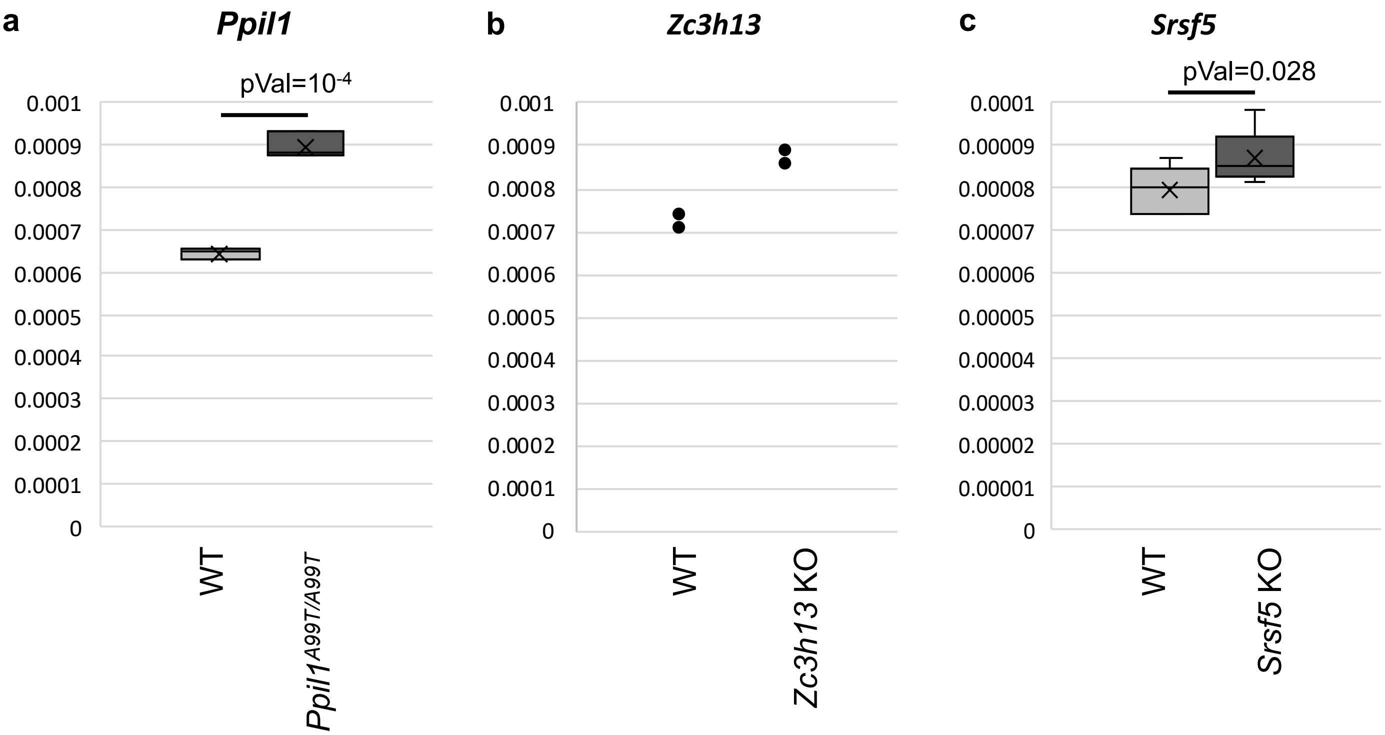

Supplementary Figure 11: Inactivation of several HP1γ molecular partners results in a significant increase in splicing noise: We mined the indicated RNA-seq data sets to quantify *de novo* splice junctions in each experimental condition. The indicated values are numbers of *de novo* junctions documented by at leads one read, normalized by the total number of reads present in each RNA-seq experiment. (**a**) E14.5 brains from WT or *Ppil1*$^{A99T/A99T}$ knock - in mice (PRJNA669300). N= 3 for each condition. (**b**) WT or *Zc3h13* KO mouse embryonic stem cells (GSE145309). N=2 for each condition. (**c**) E18.5/P0 hearts from WT or *Srsf5* KO mice (PRJNA708182). N= 6 for each condition. Boxplot center indicates the median, the (x) indicates the mean, bounds of the box indicate the 1st and 3rd quartiles, lower and upper whiskers define minima and maxima. Student's t test (two-sided unpaired). Source data are provided as a Source Data file.

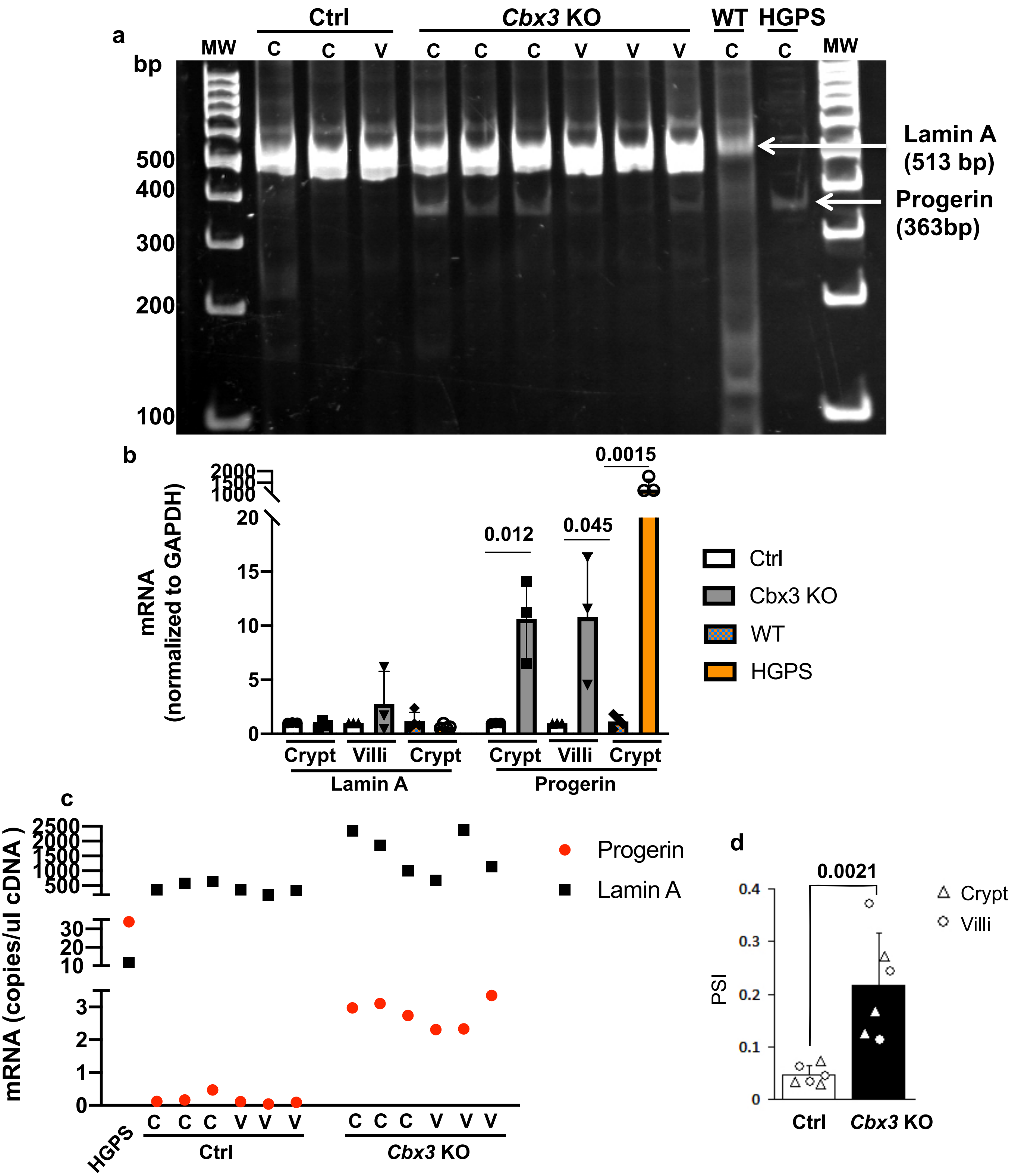

Supplementary Figure 12: Analyses of lamin A and progerin mRNA expressions upon *Cbx3* inactivation in the gut epithelium.

(a) RT-PCR using primers nested on exon 9 and 12 for detection of laminA (513 bp) or progerin (363 bp) in cDNA from crypt or Villi (V) epithelium from Ctrl, *Cbx3* KO, and 12 months aged-matched WT and HGPS G609G mice crypt epithelium, representative of n=3/4 mice per group.

(b) Taqman qPCR laminA and progerin at crypt or villi epithelia in ctrl (n=3), *Cbx3* KO (n=3) and 12 months aged-matched crypt epithelium from WT and HGPS G609G mice (n=4mice/group); in **(c)** quantification by droplet digital PCR (ddPCR) of progerin and laminA mRNA copies/ul input cDNA in crypt **(c)**, villi epithelia (V) (n=3 =mice/group) and HGPS crypt epithelium.

(d) The percentage of progerin over the sum of both transcripts (PSI, Percent-Spliced-In) was calculated with individual points represented for crypt and villi epithelia from Ctrl and Cbx3 KO mice.

Data are presented as the mean ± SEM, with Student's *t* test (two-sided). Source data are provided as a Source Data file.

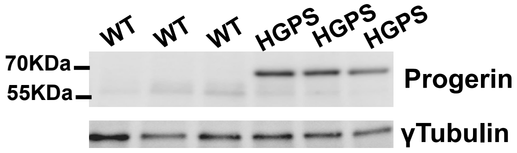

Supplementary Figure 13: Progerin expression Progerin expression was detected by Immunoblot in colon epithelium lysates derived from 12 months old WT and heterozygote HGPS G609G mice (n=3 mice in each group). Source data are provided as a Source Data file.

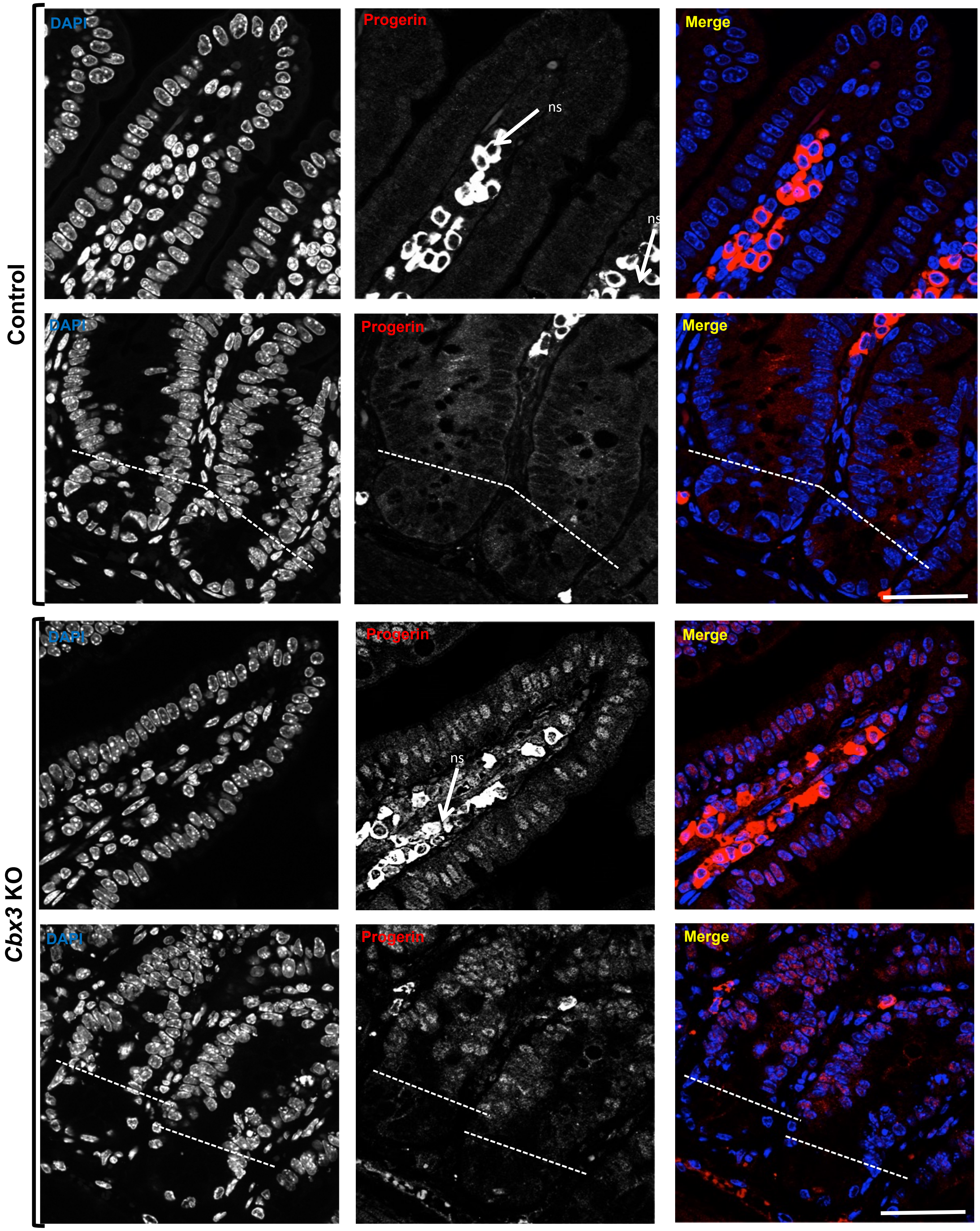

Supplementary Figure 14: Representative immunostaining of progerin in the ileon crypt and villi epithelia: progerin signal (red) is detected in the nucleus (Dapi, blue) *ns= non specific labeling (detectable with secondary antibody alone), dashed line showing the stem cell compartment at the intestinal crypt (position 0 to +4) (Scale bar: 80µm). Staining is representative of at least n=5 mice/group.

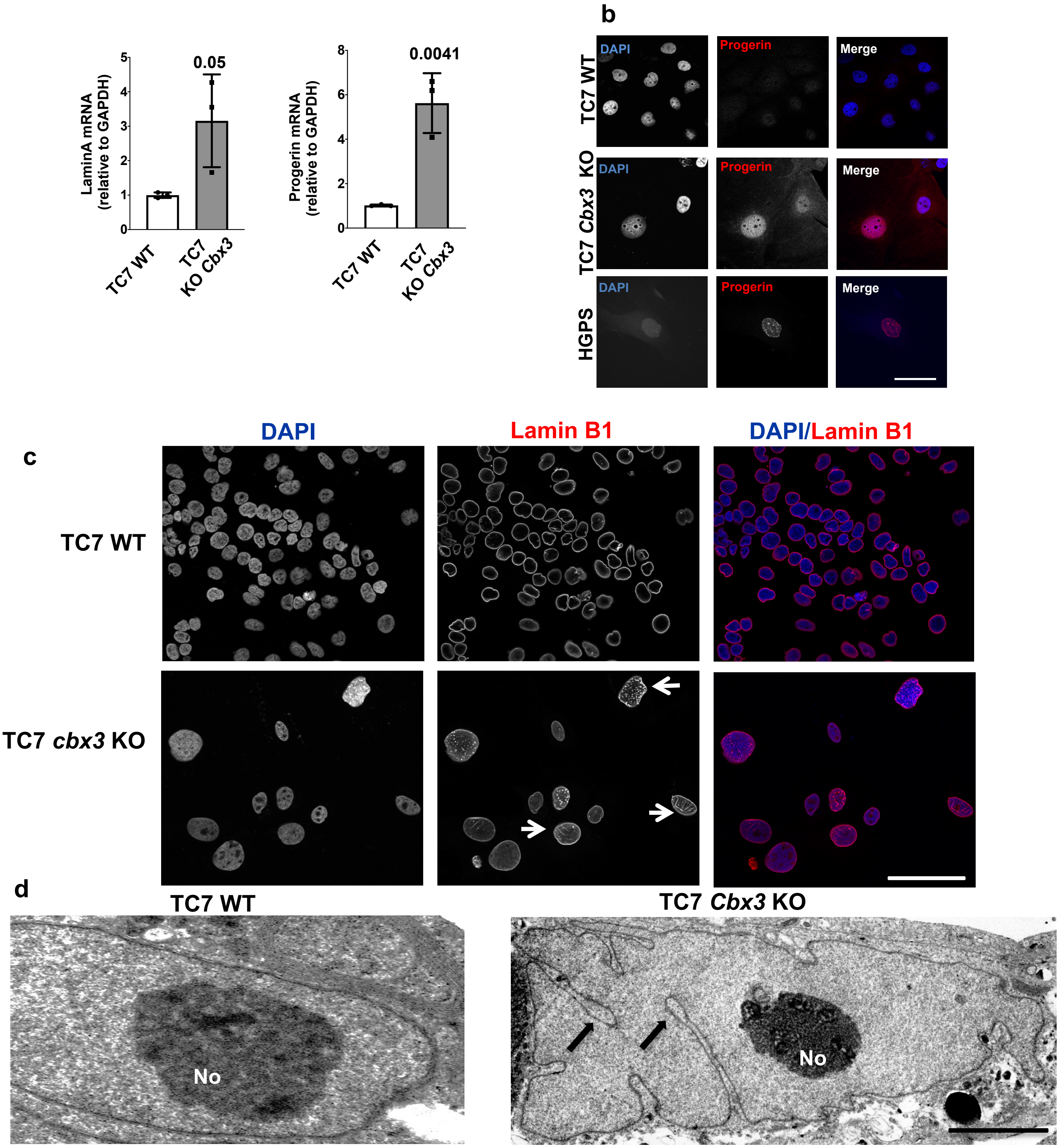

Supplementary Figure 15: *Cbx3* inactivation leads to a laminopathy with progerin production in the Crispr/Cas9 *Cbx3* enterocytic cell line TC7 (TC7 *Cbx3* KO). (**a**) RT-qPCR lamin A and progerin in the WT and Crispr/Cas9 *Cbx3* enterocytic cell line TC7 (TC7 *Cbx3* KO). Values are represented by the mean with SD of 3 independent experiments, Data are presented as the mean ± SEM; two-sided Student's *t* test (**b**) Representative immunofluorescence with anti-progerin antibody (red) and Dapi (blue) in WT, TC7 *Cbx3* KO and human HGPS fibroblast **(c)** Immunostaining with anti-lamin B1 antibody: laminB1 signal (red) reveals mis-shaping at the nuclear envelop (white arrow) reminiscent of progeria cells (Scale bar: 50µm. (b) and (c) are representative images of 3 different clones of TC7 Cbx3 KO cells, from 2 independant replicates(**d**) Transmission Electron Microscopy (TEM) on TC7 cells (WT and *Cbx3* KO). The nuclear envelop shows multiple invaginations in TC7 *Cbx3* KO cells (black arrows). Images are representative of 3 independant experiments. No = Nucleolus. (Scale bar: 2µm WT, 5µm *Cbx3* KO). Source data are provided as a Source Data file.

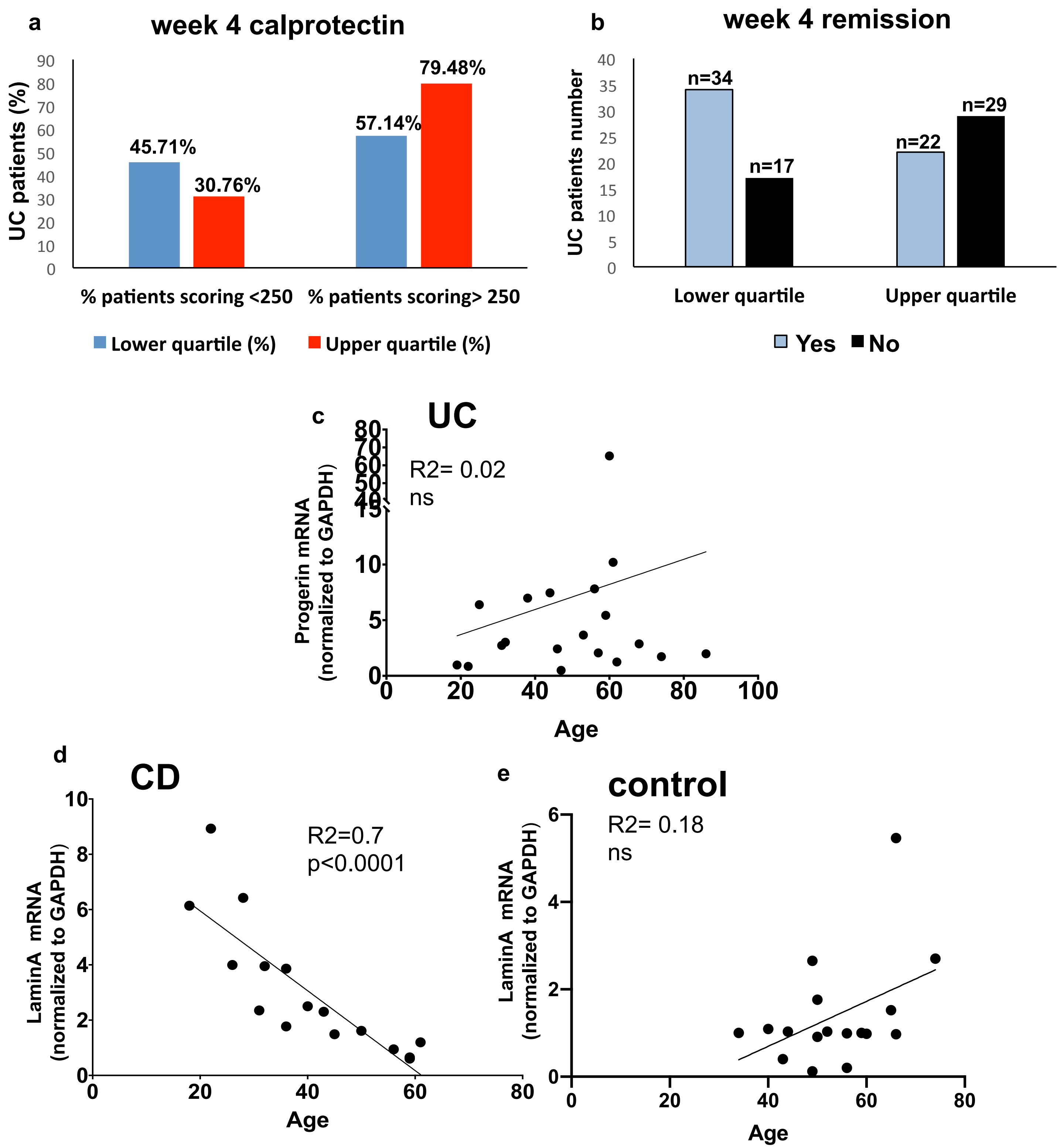

Supplementary Figure 16: (a-b) For the upper or lower quartile, histograms report patients in (**a**) with a fecal calprotectin level either below or above 250mcg/gm at week 4 (indicative of mucosal healing) and in (**b**) patients scoring "yes" and " no" for "Week 4 clinical remission", based on the metadata from the PROTECT cohort.
(**c**) progerin mRNA expression in UC (ulcerative colitis) patients relative to the age (**d-e**) laminA mRNA expression relative to the age in (**d**) CD (Crohn's disease) and (**e**) control patients. Statistical analyses was performed by simple linear regression using Prism 8 (**c-e**). Source data are provided as a Source Data file.

Supplementary Table 1: Primers used for alternative splicing (related to Suppl. Fig 9 and 10)

Name	*Sequence (5'->3')*	*Used for*
**GAPDH**		
mGAPDH-F	TGCAGTGGCAAAGTGGAGAT	RT-qPCR
mGAPDH-R	ACTGTGCCGTTGAATTTGCC	
**CD44**		
mCD44-C5-F	TTGTCAACCGTGATGGTACTCGCT	RT-PCR /gel
mCD44-v6-R	TCTGTCACATGGGAGTCTTCACTTGG	
mCD44-v5-F	TCAGCACCAGTGCTCATGGAGAAA	RT-PCR /gel
mCD44-C16-R	ATCCATGAGTCACAGTGCGGGAA	
mCD44-C2-F	TTTGAATGTAACCTGCCGCTACGC	RT-qPCR
mCD44-C2-R	AGGTACTGTTGAAAGCCTGGCAGA	
mCD44-v3-F	ACGGAGTCAAATACCAACCCAACAGG	RT-qPCR
mCD44-v4-R	ATCCTGGTGGTTGTCTGGAGTAGT	
mCD44-v4-F	TTTCTGCCCGCACAGAAGACAA	RT-qPCR
mCD44-v5-R	GGTTATTGAAAGGAGGCTGTGGTTCC	
**TCF7**		
mTCF7-e6e7F	GCCCCCTATCCCAGGTTCAC	RT-qPCR
mTCF7-e7R.94	TCTATCATATGGCTGCAGCTCC	inclusion of exon6
mTCF7-e5e7F	CATACTGTGAGCTGGTTCACC	RT-qPCR
mTCF7-e7R.94		skipping of exon6
mTCF7-e8e9F	CAGGTGGCATGCACTATCTC	RT-qPCR
mTCF7-e9e10R	CTTCTTTCCGTAGTTATCCCGC	
**PKM**		
mPKM-e8e9F	ATGCAGCACCTGATAGCTCG	RT-qPCR PKM1
mPKM-e9R.137	TCCGTGAGAACTATCAAAGCTG	inclusion exon9
mPKM-e10F.27	CTACCACTTGCAGCTATTCGAG	RT-qPCR PKM2
mPKM-e11R.42	GATTTCGAGTCACGGCAATGA	inclusion exon10
**MUC13**		
mMUC13-e2F.53	GAGAGGACACATTTCCTCCTTG	RT-PCR /gel
mMUC13-e3R.128	AGGCATTTCTTCTTCATGGTGA	intron analysis
mMUC13-i3F.2393	GCTTTGAACTTGATAGGGAGCA	RT-PCR /gel
mMUC13-i4e5R	AACTACCTGTAGACAGAGTCCC	intron analysis
mMUC13-i4F.191	ATTTTGTTATGCTGAGAAGGAGCC	RT-PCR /gel
mMUC13-i4e5R		intron analysis
mMUC13-e2F.53		RT-qPCR / gel
mMUC13-e4e5R	ATGAACTACCTGTGGAGAGCT	inclusion of cryptic exon4
mMUC13-e2e3F	TTTTGGTGACCGTGGATTGG	RT-qPCR
mMUC13-e4e5R		inclusion of cryptic exon3 & exon4
mMUC13-e3F.114	GTGATTTTCTGGAGTCACCATGA	RT-qPCR
mMUC13-e4R.91	AGCTAGCTGAGTGTAGAGTTGT	level of cryptic exon3 & exon4
mMUC13-e6F.22	CTAACCCCTGTAAAGGAACTGC	RT-qPCR
mMUC13-e6e7R	GGAATGTCGTTCCTTTCACACA	level of MUC13
