# Supplementary material for: The Heterochromatin protein 1 is a regulator in RNA splicing precision deficient in ulcerative colitis
Source: Nat Commun. 2022 Nov 18;13:6834. doi: 10.1038/s41467-022-34556-3 (PMC9674647; doi:10.1038/s41467-022-34556-3)
Supplement: Supplementary file 3 — Description of Additional Supplementary Files [file 41467_2022_34556_MOESM3_ESM.pdf]

**Description of Additional Supplementary Files**

File Name: Supplementary Data 1
Description: Patient Characteristics

File Name: Supplementary Data 2
Description: RNA seq analysis using GSEA in crypt, villi and colon epithelia comparing *Cbx3* KO to Ctrl mice

File Name: Supplementary Data 3
Description: Anti-microbial peptide gene expression extracted from the RNA seq data in crypt, villi and colon epithelium significantly modified upon *Cbx3* inactivation

File Name: Supplementary Data 4
Description: Statistical analyses of the beta diversity in Villin-Cre *Cbx3* KO male and female 7 days post tamoxifen administration

File Name: Supplementary Data 5
Description: 16S rRNA illumina MiSeq data showing the OTU significantly modified upon *Cbx3* inactivation in male and female mice feces ay 7 post-tamoxifen administration

File Name: Supplementary Data 6
Description: List of interactants detected by mass spectrometry upon Immunoprecipitation of endogenous HP1□ in HeLa cells (3 biological replicates, Control/Ctrl IgG versus anti-HP1g antibody)

File Name: Supplementary Data 7
Description: Differential splicing events (FDR<0.05) categorized by rMATS at the crypt

File Name: Supplementary Data 8
Description: Differential splicing events (FDR<0.05) categorized by rMATS at the villi

File Name: Supplementary Data 9
Description: Differential splicing events (FDR<0.05) categorized by rMATS at the colon

File Name: Supplementary Data 10
Description: Genes subject to modified splicing noise upon inactivation of *Cbx3*, while unaffected in their expression level

File Name: Supplementary Data 11
Description: Percentage splicing index (PSI) scores of the progerin-specific splicing event for individual crypt and villi epithelial samples

File Name: Supplementary Data 12
Description: Metadata from GSE109142 associated with splicing noise score (number of de novo junctions/bases in RNA-seq data)

File Name: Supplementary Data 13
Description: Splicing noise in UC patients: a gene-per-gene analysis

File Name: Supplementary Data 14
Description: Lamin A, progerin and Cbx3 mRNA expression in the IBD cohort
