# Supplementary material for: The Heterochromatin protein 1 is a regulator in RNA splicing precision deficient in ulcerative colitis
Source: Nat Commun. 2022 Nov 18;13:6834. doi: 10.1038/s41467-022-34556-3 (PMC9674647; doi:10.1038/s41467-022-34556-3)
Supplement: Supplementary file 18 — Reporting Summary [file 41467_2022_34556_MOESM18_ESM.pdf]

nature portfolio

Corresponding author(s): *Double-blind peer review submissions: write DBPR and your manuscript number here instead of author names.*

Last updated by author(s): *YYYY-MM-DD*

Reporting Summary

Nature Portfolio wishes to improve the reproducibility of the work that we publish. This form provides structure for consistency and transparency in reporting. For further information on Nature Portfolio policies, see our Editorial Policies and the Editorial Policy Checklist.

Statistics

For all statistical analyses, confirm that the following items are present in the figure legend, table legend, main text, or Methods section.

n/a	Confirmed	
☐	☒	The exact sample size (*n*) for each experimental group/condition, given as a discrete number and unit of measurement
☐	☒	A statement on whether measurements were taken from distinct samples or whether the same sample was measured repeatedly
☐	☒	The statistical test(s) used AND whether they are one- or two-sided<br>*Only common tests should be described solely by name; describe more complex techniques in the Methods section.*
☐	☒	A description of all covariates tested
☐	☒	A description of any assumptions or corrections, such as tests of normality and adjustment for multiple comparisons
☐	☒	A full description of the statistical parameters including central tendency (e.g. means) or other basic estimates (e.g. regression coefficient) AND variation (e.g. standard deviation) or associated estimates of uncertainty (e.g. confidence intervals)
☒	☐	For null hypothesis testing, the test statistic (e.g. *F*, *t*, *r*) with confidence intervals, effect sizes, degrees of freedom and *P* value noted<br>*Give P values as exact values whenever suitable.*
☒	☐	For Bayesian analysis, information on the choice of priors and Markov chain Monte Carlo settings
☒	☐	For hierarchical and complex designs, identification of the appropriate level for tests and full reporting of outcomes
☒	☐	Estimates of effect sizes (e.g. Cohen's *d*, Pearson's *r*), indicating how they were calculated

Our web collection on statistics for biologists contains articles on many of the points above.

Software and code

Policy information about availability of computer code

Data collection	ZEN software for ZEISS microscope (Apotome, Light Sheet, Transmition Electron Microscopy), BioRad Image Lab Software (Version 6.1), MxPro qPCR Software (Version MX3000p).
Data analysis	Fiji (https://fiji.sc/) ImageJ 1.53c version; PRISM 10 for Windows (Version 10.01); Perseus software version 1.6.7.0 for proteomic data (www.perseus-framework.org); For microbiota data: richness and diversity indexes using the Phyloseq package (v 1.19.1) in RStudio software and for the heatmap, a negative binomial model was fit to each OTU, using DESeq2 according to Love et al, 2014 (doi: 10.1186/s13059-014-0550-8) additional informations provided in materiel and methods; for RNAseq : rMATS (v4.1.0) for differential alternative splicing events analysis; for analysis of de novo junctions, junctions de novo were retrieved from BAM files using regtools (https://github.com/griffithlab/regtools), Randomized junctions were generated with the bedtools suite (v.2.25.0), for splice sites strength: MaxEnt algorithm (MaxEntScan::build)

For manuscripts utilizing custom algorithms or software that are central to the research but not yet described in published literature, software must be made available to editors and reviewers. We strongly encourage code deposition in a community repository (e.g. GitHub). See the Nature Portfolio guidelines for submitting code & software for further information.

March 2021

Data

Policy information about availability of data

All manuscripts must include a data availability statement. This statement should provide the following information, where applicable:

- Accession codes, unique identifiers, or web links for publicly available datasets
- A description of any restrictions on data availability
- For clinical datasets or third party data, please ensure that the statement adheres to our policy

All data are available from the corresponding author upon reasonable request. The information and requests for resources and materials should be directed and will be fulfilled by Dr. Laurence Arbibe (laurence.arbibe@inserm.fr). The source data underlying all figures in the main text of the manuscript are provided as a Source Data file.

Human research participants

Policy information about studies involving human research participants and Sex and Gender in Research.

Reporting on sex and gender	The characteristic of the 2 populations used in this study included age, gender, disease activity and duration, medications are detailed in Supplementary data table 1 and Supplementary data table 12. QPCR data for each patient are also included in the Supplementary data table 12.
Population characteristics	see above
Recruitment	Written informed consent was obtained from all individuals for the clinical and biological collection. Biopsies were performed in non-inflamed area in the right or the left colon to allow a comparison with the healthy tissue in control patient. Samples from control and active and non active IBD patients were collected without anyself selection or bias
Ethics oversight	The protocol was approved by the local Ethics Committee (CPP-Ile de France IV No. 2009/17, and No2014-A01545-42)

Note that full information on the approval of the study protocol must also be provided in the manuscript.

Field-specific reporting

Please select the one below that is the best fit for your research. If you are not sure, read the appropriate sections before making your selection.

☒ Life sciences ☐ Behavioural & social sciences ☐ Ecological, evolutionary & environmental sciences

For a reference copy of the document with all sections, see nature.com/documents/nr-reporting-summary-flat.pdf

Life sciences study design

All studies must disclose on these points even when the disclosure is negative.

Sample size	No statistical method was used to predetermine sample size. Experiments were independently repeated as indicated, and mean and the standard error from the mean were calculated. In studies with animals, the animal numbers were chosen to reflect the expected magnitude of response taking into account the variability observed in previous experiments. For the two clinical studies, the patients were selected to avoid any gender or age bias. No specific sample size calculation was made due to the exploratory nature of our study. Enrollment of all the patients was performed at the IDB gastroentrology Unit at the Beaujon, hospital, Paris.
Data exclusions	No data were excluded from the analyses.
Replication	Experiments were carried out in biological and/or technical replicates as systematically indicated in the results part (text and figure legends). The reproducibility of the experimental findings were verified by performing additional independent experiments (at least three) or by having several technical replicates (as described in the figure legends). All attempts at replication were confirmed to be successful.
Randomization	In the mice study, due to the fact of having a limited mice number, we used nonprobability sampling where arbitrary individuals were selected. Since the animals lived in the same environment, had the same food and were all siblings, randomization was not necessary.<br>The 2 human clinical studies were retrospective, and thus non randomized
Blinding	The data analysis or imaging experiments were carried out by blinded manner, applying numerical codes to the samples, verified once the experiment was concluded to try not to influence the researcher's criteria. All mice, human tissue samples and cell extracts were inventoried using randomly generated codes, unknown to the investigator at the time of conducting the experiments. In this way, the experimenter would not be in any way suggestible.

Reporting for specific materials, systems and methods

We require information from authors about some types of materials, experimental systems and methods used in many studies. Here, indicate whether each material, system or method listed is relevant to your study. If you are not sure if a list item applies to your research, read the appropriate section before selecting a response.

Materials & experimental systems

n/a	Involved in the study
☐	☒ Antibodies
☐	☒ Eukaryotic cell lines
☒	☐ Palaeontology and archaeology
☐	☒ Animals and other organisms
☐	☒ Clinical data
☒	☐ Dual use research of concern

Methods

n/a	Involved in the study
☒	☐ ChIP-seq
☒	☐ Flow cytometry
☒	☐ MRI-based neuroimaging

Antibodies

Antibodies used

Primary: Progerin (13A4D4, sc-81611, Santa Cruz), HP1α (2H4E9, Novus Biologicals), HP1β (1MOD-1A9, Thermo Scientific) and HP1γ (2MOD-IG6, Thermo Scientific) and γTubulin (4D11, Thermo Scientific), KI67 (ab16667, Abcam), Olmf4 (D6Y5A, Cell Signalling) Brdu (MA3-071, Thermo Scientific), laminB1 (ab65986, Abcam), Nucleolin (ab22758, Abcam). Anti-γTubulin (4D11, Thermo Scientific, Catalog # MA1-850), Anti-HA (12CA5) Sigma Ref. 11583816001. anti-V5 (Bethyl A190-120A) catalog A190-120ª, anti-SRSF1 (Santa Cruz sc-33652) Monoclonal ANTI-FLAG® M2 antibody produced in mouse (Sigma, F3165-.2MG), . Alexa Fluor® 488 AffiniPure Goat Anti-Mouse IgG (H+L) Code: 115-545-003, . Alexa Fluor® 488 AffiniPure Goat Anti-Rabbit IgG (H+L) Code: 111-545-003 StarBright Blue 700 Goat Anti-Rabbit IgG, 80 µl #12004162, StarBright Blue 700 Goat Anti-Mouse IgG, 80 µl #12004159

Validation

Validation statements for all the antibodies used in the study are available at the websites of the respective commercial providers. For western blot analysis as well in immunofluorescence studies using the progerin antibody 13A4, validation have been further performed using intestinal epithelial cells from HGPS mice (Extended data Figure 7) and human fibroblasts from HGPS patients (Extended data Figure 9b). For the HP1 family, we validated their use on different tissues and in TC7, HT29MTX and Hela lines. By fluorescence microscopy, they were detected at the expected nuclear location, and by western blot, at the expected molecular weight. As described in the article, KO Cbx3 makes the HP1 signal disappearing, corroborating its specificity. Gamma Tubulin has been tested and is listed on the company's website, where 18 articles are cited in which it has been used.Ki67 has been validated on the web by KO and recombinant, demonstrated in the more than 1700 references cited. Olfm4 has been validated by recombinat in the web, cited 56 times. BrdU Antibody was verified by Cell treatment to ensure that the antibody binds to the antigen stated (Thermo webpage) with 25 references.LaminB1 was tested by abcam, data in the webpage, by positive and negative controls. They provide 22 references.Nucleolin was tested again by Abcam, demostrated on the webpage and provided 135 references on research papers. Anti-HA (12CA5) validated in more tan 100 scientific papers privided in the webpage og the product. anti-V5 (BethylA190-120A) validated by Bethyl (login needed to check that info) and certified by 79 cited papers (visible on the webpage). anti-SRSF1 (Santa Cruz sc-33652) validated by shRNA (not show non the web) but validation tested and demostrated in 52 citations on the Santa Cruz webpage.

Eukaryotic cell lines

Policy information about cell lines and Sex and Gender in Research

Cell line source(s)

TC7 cell line was obtained from Caco-2 cell line, authenticated by European Collection of Authenticated Cell Cultures (ECACC). 293T cell line (CRL-1573) were from ATCC. HeLa cell line (CCL-2) were from ATCC.

Authentication

TC7 cells authentication: the cells were authenticated by ECACC as mentioned in this document. After received, we authenticated them by morphology and studying their properties (for example, by performing in vitro differentiation tests).

293T cell line (CRL-1573) and HeLa cell line (CCL-2) were from ATCC. Commonly misidentified lines does not apply.

Mycoplasma contamination

Mycoplasma test were systemically performed for the 3 cell lines using commercial PCR Mycoplasma Detection Kit (ABM, #G-238). All tests were negative.

Commonly misidentified lines
(See ICLAC register)

Does not apply.

Animals and other research organisms

Policy information about studies involving animals; ARRIVE guidelines recommended for reporting animal research, and Sex and Gender in Research

Laboratory animals	C57BL/6 Cbx3fl/fl mice were provided by Dr Florence Cammas and crossed with Villin-CreERT2 mice to produce the Villin-creERT2:Cbx3-/- mice model in this study. The generation and genotyping of IL10/Nox1 double mutant mice are described in Treton et al, 2014 (doi:10.1371/journal.pone.0101669). All the experiments using Villin-creERT2:Cbx3-/- mouse model were performed with male of female mice 2-3 months of aged. All animals used in the study were fed ad libitum standard chow diet (Teklad global protein diet; 20% protein, 75% carbohydrate, 5% fat) and kept under 12h/12h (8am/8pm) light on/off cycle. All the experiments using Villin-creERT2:Cbx3-/- mouse model were performed with male of female mice 2-3 months of aged. The animals were kept under 12h/12h (8am/8pm) light on/off cycle, a temperature of 22ºC+/-2 and a humidity between 50-70%. All animal studies were performed by authorized users in compliance with ethical regulations for animal testing and research.
Wild animals	The study did not involve wild animals.
Reporting on sex	Our findings do not seems to be related to sex gender. However, our findings provide evidence for a stronger dysbiosis in female KO Cbx3 mice.
Field-collected samples	The study did not involve samples collected in the field.
Ethics oversight	The study was approved by the ethical committee of Paris Descartes University (authorization number 17-022)

Note that full information on the approval of the study protocol must also be provided in the manuscript.

Clinical data

Policy information about clinical studies

All manuscripts should comply with the ICMJE guidelines for publication of clinical research and a completed CONSORT checklist must be included with all submissions.

Clinical trial registration	*Provide the trial registration number from ClinicalTrials.gov or an equivalent agency.*
Study protocol	*Note where the full trial protocol can be accessed OR if not available, explain why.*
Data collection	*Describe the settings and locales of data collection, noting the time periods of recruitment and data collection.*
Outcomes	*Describe how you pre-defined primary and secondary outcome measures and how you assessed these measures.*
